# Adult mortality in sub-Saharan Africa using 2001—2009 census data: does estimation method matter?

**DOI:** 10.1186/s41118-017-0025-3

**Published:** 2018-08-01

**Authors:** Clifford Odimegwu, Vesper H. Chisumpa, Oluwaseyi Dolapo Somefun

**Affiliations:** Demography and Population Studies, School of Public Health and Social Sciences Faculty of Humanities, University of the Witwatersrand, Johannesburg, 2000 South Africa

**Keywords:** Mortality, Adult, Orphanhood, Widowhood, Siblinghood, Estimation, Direct, Indirect, Sub-Saharan Africa

## Abstract

Adult mortality is an important development and public health issue that continues to attract the attention of demographers and public health researchers. Controversies exist about the accurate level of adult mortality in sub-Saharan Africa (SSA), due to different data sources and errors in data collection. To address this shortcoming, methods have been developed to accurately estimate levels of adult mortality. Using three different methods (orphanhood, widowhood, and siblinghood) of indirect estimation and the direct siblinghood method of adult mortality, we examined the levels of adult mortality in 10 countries in SSA using 2001–2009 census and survey data. Results from the different methods vary. Estimates from the orphanhood data show that adult mortality rates for males are in decline in South Africa and West African countries, whilst there is an increase in adult mortality in the East African countries, for the period examined. The widowhood estimates were the lowest and reveal a marked increase in female adult mortality rates compared to male. A notable difference was observed in adult mortality estimates derived from the direct and indirect siblinghood methods. The method of estimation, therefore, matters in establishing the level of adult mortality in SSA.

## Introduction

Adult mortality highlights the significance of demographic transition, especially in developing countries. It is another indicator of the differences within and between developing and developed countries (Graham et al. 1989; World Bank 2012). Empirical data on the levels of mortality experienced by adults in the sub-Saharan Africa (SSA) region has supported this opinion, particularly with the focus on maternal mortality (Obermeyer et al. 2010b). The high mortality of adults in SSA is now being recognised more widely, and a response has begun to emerge, particularly with regard to the impact of the AIDS epidemic and high mortality due to tuberculosis, lower respiratory infections (LRI), and malaria (Bradshaw and Timaeus, 2006).

Demographically, adult mortality is measured by the probability of dying between the ages of 15 and 60 years. There has been a substantial decline from a global average of 198 deaths per 1000 persons alive at age 15 and above between 1990 and 1995 to 157 deaths per 1000 between 2010 and 2015 (United Nations 2013). However, this rate has stalled somewhat, particularly in developing countries. Nonetheless, variations exist in the level of adult mortality among different countries in SSA. For example, the adult mortality rate in South Africa has been estimated at 43%, compared to 37% in Nigeria and 27% in Kenya (United Nations 2013). Research has documented that the high adult mortality in South Africa may be due to the HIV/AIDS pandemic which has mostly affected the Southern part of Africa.

Levels and trends in adult mortality are important indicators of development in the health of populations, and synthetic measures of the level of mortality, such as life expectancy at birth or at age 15 and above, are used as indicators of health status and social development (Helleringer et al. 2014; Timæus 1991a, 1992, 2013c). However, these are best monitored through comprehensive vital registration systems. Reliable information on mortality is essential to the development of national and international health policies. Medically certified information is available for less than 30% of the estimated 50.5 million deaths that occur each year worldwide (Murray and Lopez 1997, 2013). The need for more accurate mortality data for many practical and research purposes is essential in the field of demography, social sciences, and epidemiology. Yet, only a minority of the world’s countries have complete vital registration systems, and demographic surveillance systems are only occasionally feasible in a few isolated areas (Gakidou and King 2006). Adequately functioning systems that produce statistics on adult mortality on a regular basis exist in only about one-third of all countries of the world (Rao et al. 2006). In SSA, very little information has been available on adult mortality, let alone data from civil registration systems, demographic surveillance systems (DSS), census, and demographic and health surveys.

Civil registrations have been described as an administrative system used to document major events and their characteristics (particularly births and deaths). Some of the uses of the civil registration system include providing people with important documentation required to establish legal identity and family relationships, make claims of nationality, exercise civil and political rights, access services, and participate in modern societies (AbouZahr et al. 2015). In addition, records of vital events from civil registration are a key source of vital statistics for fertility and mortality. A working vital registration system can help furnish governments with reliable and up-to-date population and mortality statistics, including causes of death. This enables them to plan, deliver, and effectively monitor health and social development programmes, and track progress towards international commitments such as the Sustainable Development Goals (SDGs). Vital registration systems cover only a small fraction of deaths in most parts of SSA. With the notable exceptions of South Africa, Mauritius, Zimbabwe, and North African countries, it is often the case that less than 25% of deaths are recorded (Masquelier et al. 2014). As a result, adult death rates are routinely estimated based on extrapolations from child mortality rates and model life tables (Masquelier et al. 2014). However, it is important that empirical approaches are used to study adult mortality. This is because models could be imprecise when applied to specific settings due to the fact that input data and assumptions tend to over generalise epidemiological conditions which are very unlikely to change in the short term (Ben-Shlomo and Kuh 2002).

This study focuses on estimating adult mortality rates using different methods of estimation in selected SSA countries due to the fact that information on levels and trends of adult mortality and age patterns of mortality in SSA is scarce.

The ubiquitous lack of vital registration data makes it necessary to derive estimates of adult mortality using indirect demographic techniques. Indirect methods of estimating mortality are used to fill information gaps. Also, given that adult mortality is a rare event relative to the size of the population, such approaches are considered efficient and cost-effective ways of obtaining an approximate level of adult mortality. In addition, they are usually based on survey and census data (Alam and Townend 2014; Hill et al. 2006).

The other readily available data that can be used to estimate adult mortality comes from the (Demographic and Health Surveys (DHS) (Table 4 in Appendix)); at least one of which has been conducted in most SSA countries. These provide valuable information on mortality in adulthood, as well as in childhood. Many of the surveys conducted in Africa include sibling history modules that were initially developed to obtain information for deriving estimates of maternal mortality.

Furthermore, most SSA countries now have censuses available that can be used to determine the level of adult mortality. Censuses can be used to estimate adult mortality when the census schedules include either direct questions on household deaths in the recent past or questions on the survival of parents of the household members. Therefore, sibling history data collected by nationally representative surveys and orphanhood data collected by the censuses are now primary sources of information for estimating adult mortality in SSA countries where there are limited civil and vital registration systems.

The orphanhood method is one of the oldest techniques used for estimating adult mortality from the survival of specific age categories of respondents’ parents (Brass and Hill 1973; Reniers et al. 2011; Timæus, 1991b, 1992, 2013c). It is considered the most applicable method for indirect estimation of adult mortality because the survival status of the respondent’s parents is included in many censuses or surveys. This simple and robust way of analysing data on the survival of mothers and fathers was proposed by Brass and Hill (1973).

The widowhood method can be used to estimate adult mortality, and it requires questioning ever-married adults about the survivorship of their first spouse (Hill and Trussell 1977). One problem associated with this method is that it can produce biased results in polygamous societies, as the deaths of subsequent, and often younger, wives are ignored. In addition, there is also the problem of over-reporting of a husband by co-wives. Also, husbands may intentionally omit or misreport death owing to the sensitivity of the event or to feelings of liability. Estimates obtained from the widowhood method would also naturally exclude deaths of unmarried women and men (Graham et al. 1989).

The siblinghood method can be used to estimate adult mortality directly and indirectly (Timæus 2013b). Adult mortality can be indirectly estimated from data supplied by adults on the survival of their adult siblings (that is brothers and sisters) from the age of 15. The proportion of brothers or sisters surviving by age of respondent is an indicator of survivorship by approximating the probability of survival from birth to the age of the respondents. For data on sibling survival, survey respondents are asked to list all their maternal siblings by birth order and report their survival status, age, and, if deceased, age at, and date of death. The sibling history data have been used to evaluate the mortality impact of large global health initiatives, such as the United States President’s Emergency Plan for AIDS Relief (PEPFAR) (Bendavid et al. 2012), and to estimate the number of deaths due to conflict or genocide (De Walque and Verwimp, 2010; Obermeyer et al. 2008).

The use of the siblinghood method has two main concerns: sample selection bias and reporting errors (Gakidou and King 2006; Helleringer et al. 2014; Masquelier 2013; Obermeyer et al. 2010a; Timæus 2013a, 2013b; Timæus and Jasseh 2004; Trussell and Rodriguez 1990). In the case of reporting errors, these arise where respondents may be uninformed about the existence of siblings who died before they were born or when they were young. To make up for this, it has been recently proposed that questions about deaths should be restricted to siblings who survive to age 15 years or survive to marry (Timæus 2013a).

The advantage that these methods (orphanhood, widowhood, and siblinghood) of adult mortality estimation have is that they rely on information gathered by questions that are simple and easy to answer. Despite the fact that the performance of the orphanhood method has been addressed by (Brass and Hill 1973), the widowhood method by (Hill and Trussell 1977), and the siblinghood method by (Timæus 2013b), this study is important as we aim to examine whether the performance of these methods has changed over time or remained the same. In addition, at the time of the previous assessments, only few countries were explored and there has been an increase in the number of censuses conducted, as well as an improvement in the quality of data collected by the censuses over the decades. Based on this background, the aim of this study was to compare estimates of adult mortality indices for the selected countries as a way of assessing the performance of the three different methods of estimating adult mortality.

## Data and methods

### Study setting

Demographic and epidemiological information related to adult mortality in the 10 selected study countries in SSA is summarised in Table 1 in the Appendix. The 10 selected countries including Cameroon, Kenya, Liberia, Mali, Sierra Leone, South Africa, South Sudan, Sudan, Tanzania, and Uganda. South Africa has the highest HIV prevalence rate and Sudan has the lowest. HIV/AIDS was one of the leading causes of death in the study countries, except in Mali, Liberia, and Sudan. Most of the HIV/AIDS deaths occurred in South Africa. Lower respiratory infections were causes of death in all countries, except Sudan.

### Data

We used census data obtained from the Integrated Public Use Microdata Series International (IPUMS) program and survey data from the (Demographic and Health Surveys (DHS) (Table 4 in Appendix)) program. We purposefully selected 10 countries from SSA based on the availability of a recently conducted census and survey from 2000 onwards which collected information on survivorship of parents as well as spouses (census) and siblings (DHS). The selected census rounds for the countries are Cameroon (2005), Kenya (2009), Liberia (2008), Mali (2009), Sierra Leone (2004), South Africa (2001), South Sudan (2008), Sudan (2008), Tanzania (2002), and Uganda (2002). For the DHS rounds, the countries are Cameroon (2011), Kenya (2008/09), Liberia (2013), Mali (2012/13), Sierra Leone (2013), Tanzania (2010), and Uganda (2011).

## Methods

### Orphanhood method

The orphanhood method is used to produce plausible estimates of adult mortality based on reported proportions of respondents whose mother or father is still alive (Brass and Hill 1973; Henry 1960). The method estimates mortality of adult men and women indirectly from data on the survival status of the respondents’ mothers and fathers. It is based on the notion that a respondent’s mother or father must have been alive at the time of birth of the respondent. The method relates the proportion of respondents with living mothers or fathers in two adjoining age groups to measures of life table survivorship by means of a system of weighting factors whose values depend on the mean age of childbearing (Brass and Hill 1973; United Nations (1983). In order to apply the method, either a census or a single-round survey of the population must have included the following questions: ‘Is your mother alive?’ and ‘Is your father alive?’ Mortality estimates can be derived from the answers to these questions without requiring respondents to recall the exact dates when deaths occurred or the ages at death of deceased individuals.

Since the respondents’ mothers or fathers must have been alive when the respondents were born, the duration over which they have been exposed to the risk of dying equals the age of the respondents. By allowing for the mean age at which the mothers gave birth in the study population to control for variations in duration of exposure to the risk of dying by age, it is possible to estimate the life table survivorship from age 25 to age 25 plus the number of years (*n*) (age 25 + *n*) based on the age group of the respondents (*l*_25 *+ n*_*/l*_25_) from the proportion of respondents in each age group whose mother is alive. Similarly, by adjusting for the mean age at which the fathers have children, one can predict life table survivorship of adult men from the proportions of respondents with living fathers (Timæus 2013c, p. 222). Since men tend to be older than women at the time of birth of their children, their survivorship is measured between a base age of 35 and age 35 + *n*, where *n* is again linked to the age of the respondents. If mortality has changed over time, the estimated survivorship ratios reflect the mortality rates that prevailed at a range of ages and dates.

The input data required for the maternal orphanhood method include number of respondents with mother alive (or dead) classified by a 5-year age group, total number of respondents classified by 5-year age group, number of respondents who did not know or did not state the survivorship status of their mothers, and number of births occurring in a given year classified by 5-year age group of the mother. For the paternal orphanhood method, the input data required are: number of respondents with father alive (or dead) classified by 5-year age group, total number of respondents classified by 5-year age group, number of respondents who did not know or did not declare survivorship status of their fathers, and number of births occurring in a given year classified by 5-year age group of the father.

Prior to deriving mortality estimates using orphanhood data, it is important to assess how many respondents stated that they did not know whether their mother or father was alive, or failed to answer the questions at all. If the response rate on these questions is very high, they should be excluded from the analysis. Also, comparing the responses of male and female respondents of the same age is vital. The proportion of parents that have died should not differ significantly between men and women of the same age. If the proportions vary among older respondents, it could be attributed to sex differences in the pattern of age misreporting. Alternatively, it could indicate that the sex reporting fewer deceased parents (usually the men) is more likely to lose touch with their families and therefore information is wrongly captured assuming that some parents remain alive when they have died (Timæus 1992, 2013c; Zuberi et al. 2005).

One advantage that the orphanhood method has over methods based on questions regarding household deaths is that only censuses or large surveys can capture information on a reasonable number of deaths in households in the year before the inquiry to yield mortality estimates that are sufficiently precise to be useful, which is not the case with the method. Moreover, the method does not assume that the population is closed to migration (Timæus 1992, 2013c).

A natural limitation of the orphanhood method is that data on parents’ survival can only be collected from those of their offspring who are still alive. The survival status of adults who have no living children is not represented in the reported proportions of parents alive. Moreover, parents with more than one surviving child are over-represented in comparison to those with exactly one surviving child in proportion to the number of their surviving children. Thus, the method only produces unbiased results if the mortality of the parents is unrelated to how many of their children are alive at the time that the data are collected. In general, the selection bias that arises from breaches in this assumption is small (Palloni et al. 1984; Timæus 2013c). Another issue is the “adoption effect” whereby the new adopting parents are reported as if they were the (live) biological parents (whether consciously or not). This tends to result in an upward bias of the estimated survivorship of parents. Also, the “absentee effect” (the prolonged absence of fathers), in South Africa, for example, could lead to upward biases in paternal orphanhood mortality estimates (Udjo et al. 2005). In addition, there is a tendency for women to understate their ages and for men to overstate their ages which, to some extent, biases mortality estimates. Furthermore, the orphanhood method can only provide broad measures of the overall levels of adult mortality. The method is unable to detect short term trends or abnormal age patterns of mortality within adulthood (Timæus 1991a, 2013c), and the method yields mortality estimates that refer to dates before the census or survey was conducted. Therefore, orphanhood method estimates of adult mortality represent averages of mortality experienced over the period that the parents of respondents were exposed to the risk of dying. The method produces measures that refer further back in time compared to direct measures. It assumes a broad trend of mortality over approximately 10 to 15 years preceding the survey or census. Nonetheless, it is based on reliable information that is simple to collect from two questions asked from a household regarding the survivorship of the individual members’ biological mother and father.

We computed the survivorship of mothers by estimating the probabilities of surviving from age 25 to age 25 + *n* using Eq. (1) (United Nations 1983):

1 $$ \frac{l_f\left(25+n\right)}{l_f(25)}=W(n)S\left(n-5\right)+\left(1.0-W(n)\right)S(n) $$

where *S*(*n*) is the proportion of respondents aged from *n* to *n + 4* who declared that their mothers were alive at the time of the interview (age of children); *W(n)* is the weighting factor. We calculated the weighting factors (*W*(*n*)) by linear interpolation after locating the computed mean age for the mother/father from the tables of weighting factors for conversion of proportions of respondents with mother/father alive into survivorship probabilities in Manual X (United Nations 1983). According to Timæus (1992 p. 50), Brass and Hill do not give an explanation as to why they adopt a series of weighting factors, neither do they clarify why the paternal orphanhood method is less robust, yet the weighting factors are used to convert the proportion of respondents with surviving mothers to generate conditional probabilities of survival. As for the robustness of paternal orphanhood estimates, one of the possible reasons can be due to the “absentee effect” which is the absence of fathers from the household for prolonged periods of time, such that the reporting of their survivorship is not reliable (Noumbissi et al. 2005; Zuberi et al. 2005). Improved ways of using such data have been proposed by (Luy 2012; Timæus and Nunn 1997). Further estimation procedures based on regression models have been developed by the United Nations (2002) and other scholars (Timæus 2013c).

We computed the survivorship of fathers by adjusting the base from age 25 to age 35 to allow for the fact that men are usually older than women at the birth of their children (United Nations 1983). Although the standard equation is set to base ages 32.5 or 37.5, we used age 35 to correspond with the standard model life table (INDEPTH Network 2004) for translating the conditional probabilities of surviving into a common adult mortality index. We interpolated the weighting factors for age 35 from the table of weighting factors in Manual X (United Nations 1983: 103). We then used Eq. (2) to derive the conditional survival probabilities for fathers (United Nations 1983):

2 $$ \frac{l_m\left(35+n\right)}{l_m(35)}=W(n)S\left(n-5\right)+\left(1.0-W(n)\right)S(n) $$

The notations in Eq. (2) are as defined in Eq. (1) above.

To determine the reference period of the conditional survivorship probabilities, we used “time location” method developed by Brass and Bamgboye (1981) to estimate the reference periods and the exact date at which the person died. Translation of conditional probabilities of surviving into common indices of adult mortality rates is explained later, after the description of the widowhood and siblinghood methods.

### Widowhood method

The widowhood method originally developed by Hill and Trussell (1977) estimates adult mortality from information about the survivorship status of the respondents’ spouses. The rationale behind the method is that proportions of ever-married persons (classified by age) whose first spouse is still alive can be used to estimate adult survivorship probabilities. To establish the widowhood status of respondents, all ever-married respondents are asked whether their first spouse is still alive, with the possible answers being ‘yes’, ‘no’, or ‘do not know’ (United Nations 1983). To avoid problems of remarriage, the data collected refer only to the survival status of the first spouse of each respondent. The length of exposure to the risk of dying is estimated from the current age of the respondent and a measure of the average age at marriage for all respondents. The method assumes that mortality and nuptiality have remained constant in the recent past. The input data required for this method include number of ever-married male or female respondents with the first spouse alive or dead classified by the 5-year age group, total number of ever-married male or female respondents by the 5-year age group, number of ever-married male or female respondents who did not declare or did not know the survivorship status of their first spouse by the 5-year age group, and the singulate mean age at marriage (SMAM) for both males and females.

We derived female adult mortality estimates from the reports of male respondents on the survivorship of their first spouse. For male adult survivorship, we estimated from reports of female respondents on the survivorship of their first spouse. We used Eq. (3) to estimate the conditional survivorship probabilities for males (United Nations 1983):

3 $$ \frac{l_m(n)}{l_m(20)}=a(n)+b(n){SMAM}_f+c(n){SMAM}_m+d(n){NW}_f\left(n-5\right) $$

For conditional probabilities of female survivorship, we used Eq. (4):

4 $$ \frac{l_f(n)}{l_f(20)}=a(n)+b(n){SMAM}_f+c(n){SMAM}_m+d(n){NW}_m(n) $$

where for both Eqs. (3) and (4):

*n* is the age;

*a*(*n*), *b*(*n*), *c*(*n*), and *d*(*n*) are regression coefficients for estimating conditional survivorship probabilities obtained from provided tables, separately for males and females (United Nations 1983: 112);

*SMAM*_*f*_ is the singulate mean age at marriage for females;

*SMAM*_*m*_ is the singulate mean age at marriage for males;

*NW*_*m*_(*n – 5*) are male respondents aged *n* – 5 whose first spouse was alive at the time of the interview, divided by the total number of male respondents whose first spouse’s survivorship status was known;

*NW*_*f*_*(n – 5)* are female respondents aged *n* – 5 whose first spouse was alive at the time of the interview, divided by the total number of female respondents whose first spouse’s survivorship status was known.

We computed the singulate mean age at marriage using Hajnal’s (1953) method. Table 2 in the Appendix presents the computed SMAMs for the study countries. The time location for the mortality estimates derived from the widowhood method was estimated in the same manner as in the orphanhood method as stated earlier.

One of the advantages of the widowhood method over the orphanhood method is that it does not have the issue of the adoption effect and there is only one respondent per target person (Hill and Trussell 1977). Furthermore, women provide more reliable information about the survivorship of their first spouse than men (Hill and Trussell 1977).

The main concern in collecting data to be used with the widowhood method in the estimation of adult mortality arises from the fact that the analysis is intended to be applied to the survival of the respondent’s first spouse which helps to reduce the effects of remarriage on the data (Timæus 1991a; United Nations 1983). However, when marital unions in society are unstable, or if polygamy is widespread, it becomes difficult to determine the survival of the first spouse and there may be over-reporting of the male spouse by the wives. In addition, estimates of adult mortality or survival probabilities refer only to the ever-married population and not the whole adult population. The assumption that the survivorship of the respondent is independent of his or her spouse is therefore violated in countries with high HIV/AIDS prevalence. The method performs well in populations where marriage is nearly universal; however, the changing definition of marriage could pose as a challenge.

## Siblinghood method

### Indirect siblinghood method

The siblinghood method is similar to the orphanhood method but uses reports on the survival of siblings as opposed to the survival of parents. Information on the survival of brothers is used to estimate mortality of men and information on the survival of sisters is used to estimate mortality of women (Timæus 2013b; United Nations 2002). The method assumes that respondents’ siblings are approximately the same age, on average, as the respondents. Thus, the proportion of the siblings who survived to age 15 who are still alive is a good estimator of the conditional probability in a life table of surviving from age 15 to the current age of the respondents (Timæus 2013b). Mortality can be estimated from them without requiring respondents to recall the dates when deaths occurred or the ages at death of deceased individuals.

In order to apply the method, a census or survey must have asked adult respondents (for example, those aged 15 to 49) how many of their sisters and/or brothers survived to the age of 15, how many of them were still alive, and the number of those who had died.

To estimate adult mortality using the siblinghood method, we used DHS sibling history data for countries that collected this information from female respondents. It should be noted that not all of the study countries collected this information from both male and female respondents. For consistency, we only used sibling history information reported by female respondents.

To estimate adult female mortality, female respondents aged 15 to 49 were asked how many of their sisters lived to age 15 and how many of those sisters were either still alive or dead. Similarly, to estimate the mortality of adult men, information on the number of brothers who lived to age 15 and how many of these brothers were either still alive or dead was utilised. In both cases, those who did not answer either question were excluded from the calculations. The siblinghood method does not assume that the population is closed to migration.

We estimated the conditional survivorship probabilities between the exact age of 15 and 15 + *n*, where *n* is the upper limit of each age group of respondent. We calculated the conditional probabilities of surviving using Eq. (5) (Timæus 2013b, p. 248):

5 $$ {{}_{n-15}p}_{15}=a(n)+b(n){{}_5S}_{n-5} $$

where *n* is the age; *a*(*n*) and *b*(*n*) are regression coefficients; _5_*S*_*n* − 5_ is the proportion of siblings alive who were alive on the 15th birthday of respondents aged *n* – 5 to *n*.

The first advantage of the siblinghood method is that the relationship between the proportion of siblings alive and life table survivorship is assumed to be close and varies little between populations, irrespective of fertility and mortality conditions (Timæus 2013b). Secondly, the method is seen as efficient because there is more than one sibling per respondent and even small sample sizes may be useful. Lastly, it is assumed to perform better than other indirect methods (Timæus, 2013b).

The disadvantages of the siblinghood method include the following: if all siblings are dead, there would be no one to report on them; the death of one sibling is reported by all siblings; respondents omit siblings; unknown siblings who died before the birth of the respondents are usually not reported; and the assumption of independence of the probability of surviving of the siblings may not apply in certain environments.

### Direct siblinghood method

For the direct estimates of the siblinghood method (Timaeus 2013a), we first reproduced the adult mortality rates published in the demographic and health survey reports; that is, the probability of dying between age 15 and 50 years (_*35*_*q*_*15*_) for the 6-year period before the survey for the seven countries (Cameroon, Kenya, Liberia, Mali, Sierra Leone, Tanzania, and Uganda) that conducted a DHS from 2000 onwards. We used only sibling history data reported by female/sister siblings, since not all the countries had sibling histories reported by male/brother siblings. We computed the deaths and person-years of exposure for the siblings. Then, we calculated age-specific mortality rates which were converted to probabilities of dying using the standard life table conversion formula. Table 3 in the Appendix shows the reproduced estimates reported in the DHS reports for the respective countries. Having replicated the published estimates, we then proceeded to derive the estimates for the probability of dying between age 15 and 60 years (_45_*q*_15_).

### Translation of conditional survival probabilities into adult mortality rates

The conditional survivorship probabilities we obtained using orphanhood, widowhood, and siblinghood indirect methods for each age group were converted into a single adult mortality measure to facilitate comparison over time. We chose to measure adult mortality using the probability of dying between ages 15 and 60 years (_45_*q*_15_) because other studies (Moultrie et al. 2013; Timæus and Jasseh 2004), international organisations, and agencies (United Nations 2015; World Bank 2015; World Health Organization 2013) have used it as a summary indicator of the mortality of young and middle-aged adults. It is also suitable for comparing mortality estimates derived from the three methods with adult mortality measures from other sources (Moultrie et al. 2013).

We chose the INDEPTH Network model life tables (IMLT) for SSA to represent the age pattern of adult mortality and used them as standard life tables to translate conditional probabilities of survivorship into a common adult mortality index (_45_*q*_15_) for the orphanhood, siblinghood, and widowhood methods from constructed relational life tables. The relational model life tables allow the construction of variants of life tables that are consistent with the chosen standard life table, thereby facilitating the generation of new life tables that differ from the chosen standard (Rowland 2003).

The IMLT were specifically developed for SSA countries in order to account for HIV prevalence and other mortality causative factors peculiar to the region. They were constructed from empirical life tables from 17 demographic surveillance sites in 10 African countries for the period 1995–1999 (INDEPTH Network 2004). As recommended by the INDEPTH Network (2004), for countries with a high HIV prevalence rate of 10% and above, pattern 2 of the IMLT was adopted as the standard life table for translating conditional survival probabilities into adult mortality estimates. South Africa was the only country in the analysis with a HIV prevalent rate above 10%. We used pattern 1 of the IMLT for the other countries as their HIV prevalence rates were below 10%. The logits of the conditional survivorship probabilities were calculated as:

(Timæus 2013c): $$ {Y}_x=\frac{1}{2}\ln \left(\frac{1-{{}_nP}_x}{{{}_nP}_x}\right) $$ (6)

and for the standard life table

(Timæus 2013c): $$ {Y}_x^s=\frac{1}{2}\ln \left(\frac{1-{{}_nP}_x}{{{}_nP}_x}\right) $$ (7)

where *Y*_*x*_ is the logit for the study population; ${Y}_x^s$ is the logit for the standard model life table; and _*n*_*P*_*x*_ is the conditional survivorship probability from age *x* to *x + n.*

We then used the estimated conditional survivorship probabilities to calculate the values of *α* (level of mortality) for a system of relational model life table, and this is translated into probabilities of dying between ages 15 and 60 by fitting a 1-parameter model computed for each corresponding age group. We used Eq. (8) to compute the level of mortality *α* (Timæus 2013c):

8 $$ \alpha =-\frac{1}{2}\ln \left(1+\frac{\frac{{}_nP{}_x}{l_{x+n}^s}-\frac{1}{l_x^s}}{1-{}_nP{}_x}\right) $$

where ${l}_x^s$ are values from the standard life table and _*n*_*P*_*x*_ is as defined above. The higher the values of *α*, the higher the level of mortality.

We estimated the probability of dying between age 15 and 60 years using Eq. (9) (Timæus 2013c) after fitting the relational model life table:

9 $$ {{}_{45}q}_{15}=1-\left(\frac{1+{\exp}^{2\left(\alpha +{Y}_{(15)}^s\right)}}{1+{\exp}^{2\left(\alpha, +,{Y}_{\left(15+45\right)}^s\right)}}\right) $$

The time reference for the siblinghood method was estimated differently from the orphanhood and widowhood method, and we used Eq. (10) (Timæus 2013b, p. 249):

10 $$ T=a(n)-b(n)\ln \left({{}_5S}_{n-5}\right) $$

where *T* is the time location for each conditional survivorship probability for each age group. The other terms, *a*(*n*), *b*(*n*), and _5_*S*_*n* − 5_, are as defined earlier.

### Data assessment

Data were analysed using STATA 12 and Microsoft Excel software packages. STATA 12 was used for data management to produce raw data tables that were then exported to Microsoft Excel spreadsheets for demographic assessment and analysis with orphanhood, siblinghood, and widowhood methods of indirect estimation of adult mortality. For data quality assessment, that is the reporting of survivorship of mothers and fathers as well as spouses, we computed proportions of survivorship to assess the consistency of the information collected by sex.

Census data for all the study countries were assessed and evaluated for errors, and appropriate adjustments were made, where necessary, using standard demographic procedures^1^ (Arriaga et al. 1994; Siegel et al. 2004; United Nations 1955, 1983, 2002). We assessed the quality of the data for the number of births in the last year; data on fathers/mothers alive and dead; DHS data on sibling brothers/sisters alive and dead; and spouses widowed/not widowed. In addition, census data for the orphanhood and widowhood data were weighted for all the countries except Kenya and Uganda. The data for Kenya and Uganda were not weighted because they exist as a 10% sample of census data and did not require to be weighted as recommended by IPUMS. All the DHS sibling history data were weighted with the supplied sampling weights. The number of births in the last year is an important indicator as it is used to estimate *M*, the mean age of mothers or fathers at the birth of their children. Table 2 in the Appendix presents the mean age (*M*) at child birth for both male and females, calculated for all the countries analysed, except Mali and Liberia which did not have data. However, estimates were obtained from the United Nations Population Division (United Nations 2015) (see Table 2 in Appendix).

We compared the derived adult mortality rates with those of the World Health Organization, United Nations Population Division and Reniers et al. (2011). This was done for purposes of assessing the performance of the methods and not as direct comparison of results since the other sources used different methods and data. The World Health Organization (2016) adult mortality estimates are for the period 1990–2013 and were derived from life tables constructed using United Nations World Population Prospects data. Whereas, the United Nations Population Division adult mortality estimates are for the period 1990–2010 and were derived from life tables based on smoothed projected population by age and sex data for the World Population Prospects (United Nations 2014). The adult mortality rates by Reniers et al. (2011) cover the period 1990–2005 and were estimated using sibling history data from demographic and health surveys for the respective countries and applying regression methods.

## Results

**Fig. 1:**
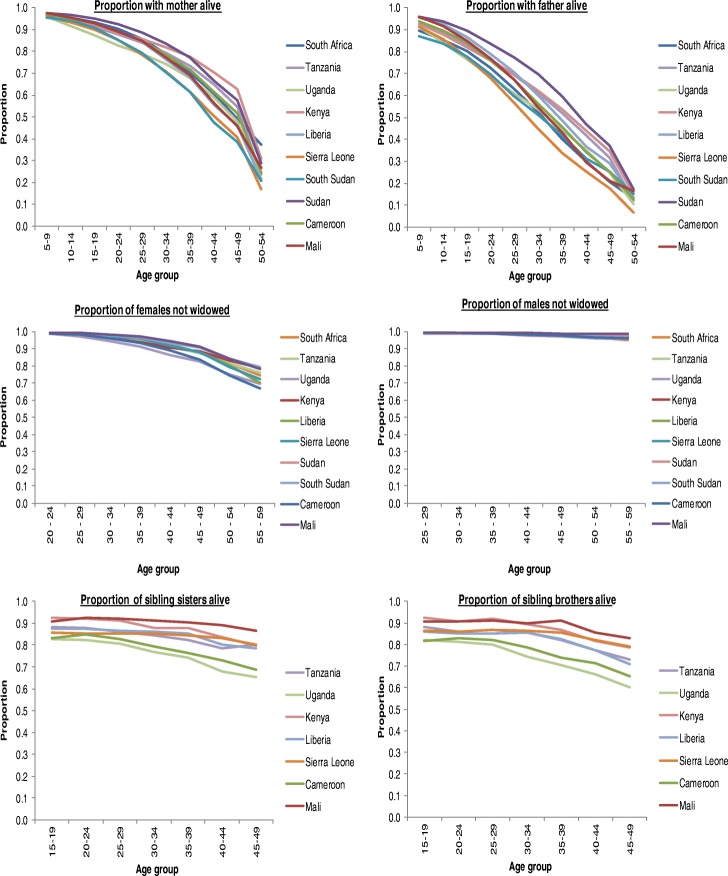
Proportions of fathers/mothers alive, spouses not widowed, and brothers/sisters alive

Studies show that women report lower proportions of surviving parents than men (Moultrie et al. 2013). Our plots of the proportion of survivorship of parents as well as spouses follow the expected pattern. For orphanhood, the proportion of survivorship for parents declined progressively with an increase in age. The proportion of sibling brothers and sisters alive slightly declines with advancing age, for siblinghood method. For widowhood, the survivorship of spouses declined progressively with age for females, but not males (an indication of problems with the data), as shown in the Fig. 1 below.

### Adult mortality estimates

The adult mortality estimates derived from the orphanhood, widowhood, and siblinghood methods have been compared with external sources whose estimates are presented in Tables 5, 6, and 7 in the Appendix. The estimates from external sources were computed using different methods from those of the study. The basis of comparison is that all the adult mortality estimates are translated to a common adult mortality index, which is the probability of dying between ages 15 and 60 years.

**Fig. 2:**
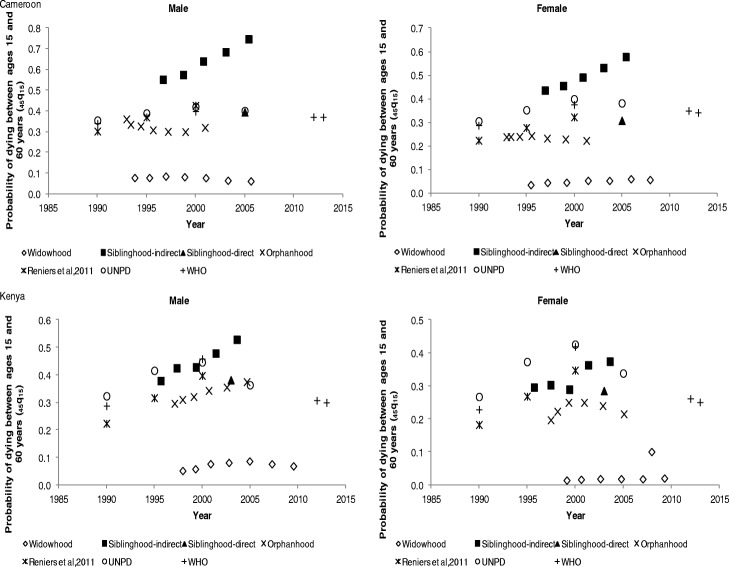
Probability of dying between ages 15 and 60 years (_*45*_*q*_*15*_) by sex in Cameroon and Kenya

Figure 2 shows the probability of dying between ages 15 and 60 years for males and females in Cameroon and Kenya. Generally, adult mortality increased from the mid-1990s to early 2000s; also considered the peak of the HIV/AIDS epidemic. From mid-2005, however, a decline in mortality is observed as shown by UNPD and WHO estimates. For Cameroon, male mortality is higher than female mortality. The orphanhood mortality estimates show that the probability of dying for males declined from 34% in the 1990s to 31% in early 2000. A similar trend is observed for females, as mortality rates declined from 23 to 22% in the same period.

The indirect siblinghood mortality estimates are (unexpectedly) the highest, whilst the widowhood estimates are the lowest, but indicate that mortality is higher for males than females. Adult mortality estimates by the UNPD, WHO, and Reniers et al. (2011) are consistent. The direct siblinghood mortality is also consistent for males. For females, minor differences are observed. Orphanhood mortality estimates are lower than the UNPD, WHO, Reniers et al. (2011), and siblinghood mortality estimates, but display a consistent trend. However, in the case of Kenya, it is also observed that male mortality is higher than for females. Between 1990 and 2000, a general increase in adult mortality is observed for both males and females. The orphanhood mortality estimates indicate that male mortality increased from 27% in the 1990s to 37% in 2005, whereas for females, mortality increased from 16 to 21% for the same period and thereafter started to decline. The direct siblinghood and orphanhood mortality estimates are consistent with the observed trend reinforced by UNPD, Reniers et al.(2011), and WHO mortality estimates. A decline in adult mortality rates for females is observed after 2005, as shown by orphanhood, UNPD, Reniers et al., (2011), and WHO mortality estimates. The indirect siblinghood mortality estimates are unexpectedly high beyond 2005, whilst the widowhood estimates are extremely low throughout the period.

**Fig. 3:**
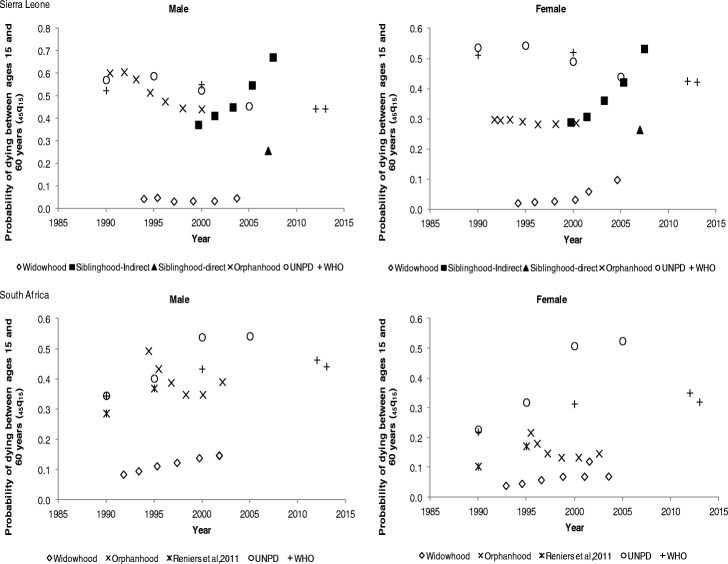
Probability of dying between ages 15 and 60 years (_*45*_*q*_*15*_) by sex in Sierra Leone and South Africa

For Sierra Leone, adult male mortality is higher than female mortality, except for the direct siblinghood estimate which indicates higher mortality for females as shown in Fig. 3. The orphanhood, UNPD, and WHO mortality estimates indicate a general decline in mortality for both females and males. However, as observed earlier, the indirect siblinghood mortality estimates are unexpectedly high after 2005. A wide gap in estimates is notable for females between 1990 and 2005 for UNPD and WHO estimates, compared to the orphanhood and direct siblinghood estimates. For South Africa, between 1990 and 2005, adult mortality increased for both males and females, before declining. The UNPD and WHO mortality estimates are higher than the orphanhood estimates for both males and females. Disparities in adult mortality rates among the methods and estimates from other sources can be observed.

**Fig. 4:**
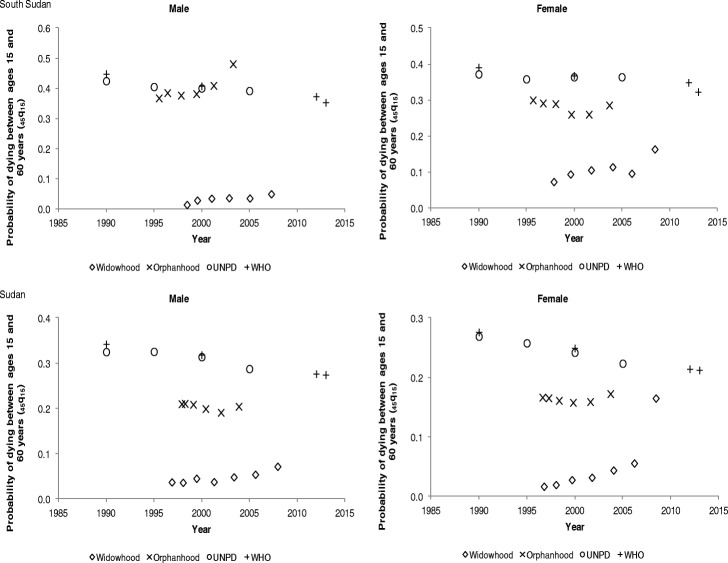
Probability of dying between ages 15 and 60 years (_*45*_*q*_*15*_) by sex in South Sudan and Sudan

For South Sudan, the orphanhood mortality estimates for males indicate a rising trend between the mid-1990s and mid-2000s whereas for females a decline and then a slight increase is observed as shown in Fig. 4. While the UNPD and WHO mortality estimates show a gradual declining trend in adult mortality for both males and females, with males experiencing higher mortality. Between 1995 and 2000, the orphanhood mortality estimates for males appear comparable to those of the UNPD and WHO. However, this is not the case for female mortality estimates. For Sudan, the orphanhood mortality estimates though lower than UNPD and WHO estimates show a gradual decline in mortality for males but a stall in female mortality. Equally, a gradual decline in adult mortality is observed for both males and females from the UNPD and WHO estimates. A gap is visible between orphanhood estimates and UNPD and WHO mortality estimates. Again, the widowhood mortality estimates are very low for both South Sudan and Sudan.

**Fig. 5:**
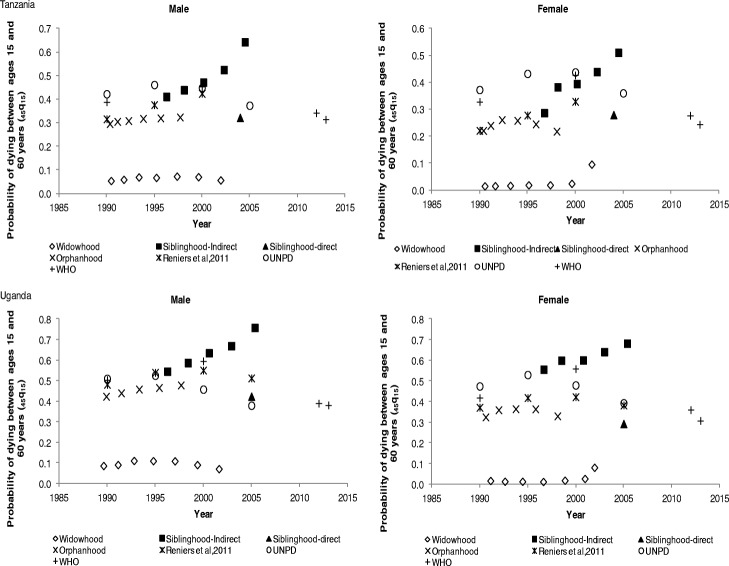
Probability of dying between ages 15 and 60 years (_*45*_*q*_*15*_) by sex in Tanzania and Uganda

In Tanzania, the probability of dying between ages 15 and 60 years is higher for males than females, as shown in Fig. 5. The orphanhood mortality estimates indicate a slight increase from 30% in 1990 to approximately 32% between 1995 and 2000, for males whilst female mortality increased between 1990 and 1995, before starting to decline by 2000. The UNPD and WHO mortality estimates indicate a gradual decline in adult mortality for both males and females. The orphanhood mortality estimates are lower than those of the UNPD and WHO but appear close to those of Reniers et al. (2011), between 1990 and 1995. The direct siblinghood mortality estimates for both males and females are slightly lower than those of UNPD. In comparison, for Uganda, adult mortality in males is higher than females. The orphanhood mortality estimates show a gradual increase in mortality from 43% in 1990 to 47% around 2000 for males, whereas female mortality slightly decreased, from 35% in 1990 to 32% in 2000. On the other hand, the UNPD and WHO mortality estimates indicate that for both males and females, mortality generally increased between 1990 and 2000, and then, started to decline after 2005. The direct siblinghood mortality estimate for males is closer to UNPD and Reniers et al. (2011) estimates. As observed earlier, the widowhood mortality estimates are the lowest, whereas indirect siblinghood adult mortality estimates rise sharply after 2005, which is unexpected.

**Fig. 6:**
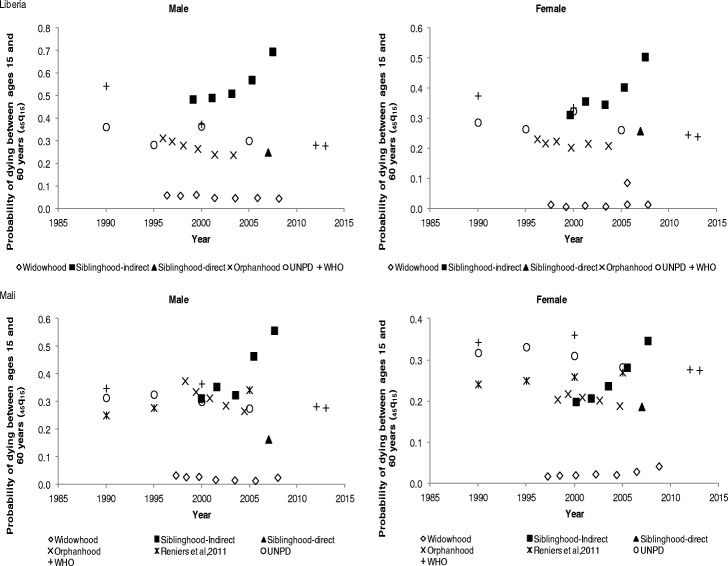
Probability of dying between ages 15 and 60 years (_*45*_*q*_*15*_) by sex in Liberia and Mali

Figure 6 shows the probability of dying between ages 15 and 60 years for males and females in Liberia and Mali. For Liberia, adult mortality is higher for males than females, except the direct siblinghood mortality estimate which shows higher mortality for females. Generally, the mortality rates indicate a declining trend for both males and females, except for indirect siblinghood and widowhood mortality estimates. The orphanhood mortality estimates show a decline from 30% in 1995 to 23% in 2005 for males, and from 25% in 1995 to 20% around 2005 for females, which is reinforced by UNPD and WHO estimates. For Mali, apart from the direct siblinghood mortality estimate which indicates higher mortality for females, the other estimates show male adult mortality is higher than female mortality. A decline in adult mortality is also observed for orphanhood estimates from 48% in 1995 to 26% in 2005 for males, whereas for females there was a decline from 23% in 1995 to 18% in 2005. The UNPD and WHO mortality estimates are consistent with the orphanhood estimates with respect to the observed pattern of the trend between 2000 and 2005 for both males and females. Between 2000 and 2005, the male orphanhood mortality rates are comparable to UNPD and WHO mortality estimates. In all cases, widowhood estimates are very low, whereas indirect siblinghood adult mortality estimates are unexpectedly higher from 2005 onwards.

Overall, adult mortality estimates derived by the orphanhood method reveal that the SSA countries generally experienced an increase in mortality for both males and females between 1990 and 2000, after which mortality began to decline.

On the other hand, the mortality estimates obtained from the widowhood method for adult mortality differ from those derived from the orphanhood and siblinghood methods and are very low.

With respect to the siblinghood mortality estimates, the indirect method estimates show that the level of adult mortality is higher for males than females, as expected. Conversely, an unexpected rising trend in the level of mortality, both for males and females, is observed across all the countries after 2005.

The direct siblinghood method mortality estimates show that Mali, Sierra Leone, and Liberia have higher levels of female adult mortality than males. Additional information on the direct siblinghood mortality estimates is presented in Table 3 in the Appendix.

## Discussion and conclusion

The aim of this study was to find out whether the use of different adult mortality estimation methods yields the same results or not. We chose to compare the orphanhood, widowhood, and siblinghood (direct and indirect) methods and applied them to selected SSA countries with available census and DHS survey data after 2000. We observed variations in the derived adult mortality estimates from the three methods.

When considering all of the methods, male adult mortality was higher than female mortality, as expected, with the exception of the direct siblinghood estimates which showed higher mortality for females in Mali, Sierra Leone, and Liberia. For the widowhood method, notable differences were observed in Mali, Cameroon, and South Sudan, where results are inconclusive. The indirect siblinghood method yielded slightly higher, yet comparable, adult mortality estimates to those derived by the orphanhood method up to 2005 for most countries. However, adult mortality estimates using the widowhood method remain lowest and underestimated for all the countries in SSA. Generally, after 2005, the indirect siblinghood method tends to show a rise in the trend of both male and female adult mortality rates which is unexpected, given the fact that a number of these countries in SSA had introduced antiretroviral therapy (ART) programmes between 2003 and 2005 to help reduce adult mortality. The possible explanation for this upward trend is that, generally, indirect methods are not sensitive to changes in mortality in the short term (Timaeus 2013b; Boerma et al. 1992). In addition, biases in the reporting of siblings contribute to the observed pattern of mortality estimates. The omission of dead siblings by older respondents biases the mortality estimates downwards at older ages, thus suggesting that adult mortality increased when compared to reports by younger siblings. Therefore, more deaths are reported in the recent period than previously. The direct siblinghood adult mortality rates were lower than those obtained from the indirect siblinghood method before smoothing. Generally, scholars (Adetunji 1996; Preston 1985) have found that indirect methods yield higher mortality estimates than direct methods. The differences have been attributed to data errors, violation of assumptions, and internal biases within the methods.

The orphanhood and siblinghood methods yielded higher adult mortality rates than the widowhood method. For example, the orphanhood and siblinghood methods were able to show that the increase in female adult mortality estimates in Uganda and Kenya may be as a result of HIV, as no other single cause of death can explain a significant rise in mortality (Dorrington et al. 2001; Tollman et al. 1999). Again, the differences in the mortality rates between the three methods could be attributed to the quality and internal reliability of the input data and assumptions used. For the widowhood method, an assessment of the proportions of spouses not widowed was consistently very high for males (above 80% across all ages), which is suspicious as proportions of spouses alive were not decreasing with age, hence leading to low mortality estimates. This indicates that the reporting of survivorship of the spouses was not reliable, particularly at older ages. This is complicated further when we consider the African context in which polygamous marriages are practiced. However, the reports of females and survivorship of their spouses seemed more reliable.

The orphanhood method uses information on survivorship of biological parents obtained across all age groups from members in the household. These data are more reliable compared to information used by the widowhood method. It is possible that individuals who remarried may not be honest in responding to questions on the survivorship status of their first spouse. For example, in South Africa, marriage is not universal and has lost its value (Chimere-Dan 1997; Swartz 2003). This is likely to cause a downward bias in the mortality estimates derived from the widowhood method. Also, in Sudan and South Sudan, mortality estimates from the widowhood method were inconclusive. We suspect that there is a possibility of poor quality of data because of the civil war.

For the siblinghood method, the sibling histories have data quality issues such as respondents underreporting dead siblings who died a very long time ago, multiple reporting of siblings, and clustering of mortality within certain sibships (selection biases). However, as Trussell and Rodriguez (1990) and Masquelier (2013) observe, these tend to cancel out leading to conservative mortality estimates.

Information on survivorship of biological parents and siblings is likely to be more reliably reported than that of survivorship of the first spouse. Hence, the differences in adult mortality estimates yielded by the methods. However, in some applications of the orphanhood method, reports about some orphans may have been referred to mistakenly for their living foster parent. When this occurs, the data on young people may be most affected (Hosegood et al. 2004). Information on parents with several surviving children risks being over-represented in the target population as survivorship estimates for young adults aged less than 20 tend to cause an upward bias in the estimated survivorship, as they may have been adopted by a relative who report themselves as the biological parent (Timæus 2013c). It is also important to note that orphanhood estimated probabilities of survivorship reflect parents with surviving children and not the whole population. The orphanhood method tends to produce smoothed mortality rates without fluctuations because they refer to long periods of time between the birth of the respondent and when the data is collected (Timæus 2013c). Whereas widowhood adult mortality estimates only apply to the ever-married population and not the whole adult population in a country.

The siblinghood adult mortality rates reflect mortality of young and middle-aged adults with respect to siblings. The siblinghood method tends to underestimate mortality for long periods of time because the older siblings omit their dead siblings. Timæus (2013a) cautions that because the sibling history information in DHS surveys is aggregated over a period of 6 or more years, the method will not reflect fluctuations in mortality rates. However, the reporting of recent deaths, especially by siblings in the age group 20–24, is assumed to be more reliable than by siblings in older age groups. Therefore, as a result, an impression is given that mortality increases overtime. The direct siblinghood method performs well when the reporting of ages and dates of deaths is complete. However, when this is not the case, the indirect siblinghood method would be preferred (Timæus 2013a). All the methods examined do not require the assumption of the population being closed to migration.

The pattern of adult mortality rates by trend also differs among the methods. The mortality trends produced by the orphanhood and direct siblinghood methods across all the study countries appeared to be more consistent than those produced by the widowhood method. The methods also performed differently from country to country. For example, the orphanhood and direct siblinghood methods appeared to perform well in Kenya, Liberia, Tanzania, and Uganda.

Despite being aware of the differences in methodology, we examined independent estimates of adult mortality from other sources such as the UNDP and WHO and found that in some cases there are consistencies, and in others, there are differences among these estimates by source. Although they are not comparable by method, our adult mortality estimates were in some cases consistent with these other external sources but also varied in other instances. We also examined siblinghood adult mortality estimates by Reniers et al. (2011) who used Poisson regression analysis to derive the rates. In terms of the trend, similar to our orphanhood and siblinghood estimates, they also showed rising mortality from 1990 to 1995 for Mali, Cameroon, Kenya, Uganda, Tanzania, and South Africa. Our siblinghood mortality estimates for Mali were comparable to those of Reniers et al. (2011) in magnitude until 2000; however, for other countries, ours were slightly higher. After 2005, our indirect sibling estimates are incomparable. This may be attributed to the differences in methods used as well as the handling of the sibship size biases.

For some time now, sufficient evidence has emerged to bring adult mortality to the attention of international and national agencies. Developing countries lack adequate vital registration systems which have posed a major challenge in the use of consistent methods for accurately estimating adult mortality. Even though substantial knowledge and ways of interpreting incomplete reports of adult mortality exist, alternative methods, such as death distribution methods, can only be applied when the majority of deaths are recorded. In addition, results from indirect estimation methods need to be interpreted carefully considering potential biases.

The study has a number of limitations; first, it is important to note that we did not adjust for the undercounting of respondents in the sibling history data when computing person-years of exposure for the direct method. Indeed, some studies (Gakidou and King 2006; Obermeyer et al. 2010a) claim that such lack of adjustment leads to an upward bias in mortality estimates. However, other studies (Masquelier 2013; Trussell and Rodriguez 1990) posit that this is offset by the cancelling out that occurs due to multiple reporting of siblings and, therefore, has minimal effect on the mortality estimates. We used the approach taken by the DHS and other studies (Reniers et al. 2011; Timæus 2013a; Timæus and Jasseh 2004) in handling the sibling history data to produce the adult mortality estimates. Furthermore, Masquelier (2013, p. 226) recommends that the standard calculation of sibling adult mortality estimation should be used on the grounds that it produces some conservative estimates.

Second, for the widowhood method, though we were required to use information on the survivorship of the first spouse, the data we used was not detailed enough to determine whether respondents were in the first or second marriage. We recognise this as a limitation of the study.

Finally, we used the INDEPTH model life tables as a standard for translating the conditional survival probabilities into a common mortality index. However, the INDEPTH model life tables are hardly representative of some of the African countries under study, and the age pattern of mortality of the model life table may not be suitable. This is because the data used in constructing these model life tables are scanty as they were taken from unrepresentative demographic surveillance sites and have not been smoothed. There is a possibility that this could also have affected our adult mortality estimates.

In conclusion, although the estimated adult mortality results produced in this study need to be interpreted carefully in light of potential biases, they are indicative of the level and trend and may not represent the actual adult mortality estimates. It is evident that the orphanhood and direct siblinghood methods yield more consistent and plausible results when compared to the widowhood method; however, based on our analysis, it is not easy to conclude that the siblinghood method is better than the orphanhood method. As previously highlighted, data quality issues (which vary from country to country in SSA), violation of assumptions, and internal biases of each method are all factors which need to be considered. Therefore, the choice of the estimation method used is important when considering adult mortality rates in SSA. Future studies should examine improvements in the indirect estimation methods in order to derive more accurate rates to be used in monitoring and evaluating progress of the SDGs, particularly goal number 3 which is to ensure healthy lives and promote well-being for all at all ages. Furthermore, investment and effort towards developing the civil and vital registration systems will enhance the availability of adult mortality data, as well as contribute to the big data revolution in SSA.

## Appendix

**Table 1:** Background information on the study countries

Country	Total population in 2015 (millions)^a^	Population growth rate (percent per annum)^b^	Birth rate (per 1000)^b^	Death rate (per 1000)^b^	Life expectancy at birth (years)^c^	Adult HIV prevalence rate in age group 15–49 (percent)^c^	HIV/AIDS deaths ^c^	Leading causes of death ^d^
Cameroon	23.7	2.5	37	11	Total: 56 Male: 56 Female: 58	4.8	34,000	HIV/AIDS, malaria and lower respiratory infections
Kenya	44.3	2.7	31	8	Total: 62 Male: 60 Female: 65	5.3	33,000	HIV/AIDS, lower respiratory infections and diarrheal diseases
Liberia	4.5	2.6	36	9	Total: 60 Male: 59 Female: 61	1.2	2000	Malaria, lower respiratory infections and diarrheal diseases
Mali	6.7	3.0	44	15	Total: 53 Male: 53 Female: 53	1.4	5300	Malaria, diarrheal diseases and lower respiratory infections
Sierra Leone	6.5	1.9	37	14	Total: 50 Male: 50 Female: 51	1.4	2700	Malaria, lower respiratory infections and HIV/AIDS
South Africa	55	0.8	22	10	Total: 61 Male: 59 Female: 63	18.9	140,000	HIV/AIDS, lower respiratory infections and diarrheal diseases
South Sudan	12.2	4	36	12	Total: 55 Male: 54 Female: 56	2.7	13,000	Lower respiratory infections, diarrheal diseases and HIV/AIDS
Sudan	40.9	2.1	38	9	Total: 62 Male: 60 Female: 64	0.2	2900	Neonatal preterm birth, congenital anomalies, schematic heart disease
Tanzania	52.3	3.0	39	9	Total: 62 Male: 60 Female: 63	5.3	46,000	HIV/AIDS, lower respiratory infections and malaria
Uganda	40.1	3.3	40	9	Total: 59 Male: 58 Female: 60	7.3	33,000	HIV/AIDS, malaria and lower respiratory infections

Sources: ^a^(Population Reference Bureau, 2015)

^b^(United Nations, 2015)

^c^(UNAIDS, 2015)

^d^(IHME, 2013)

**Table 2:** Mean age at childbearing and singulate mean age at marriage by selected sub-Saharan countries

	Census year	Mean age at child bearing (M)		Singulate mean age at marriage (SMAM)	
Country		Male	Female	Male	Female
Cameroon	2005	34.0	26.4	27.6	21.9
Kenya	2009	32.0	26.7	26.8	22.4
Liberia	2008	33.4	28.9	26.7	22.3
Mali	2009	35.6	29.4	26.4	19.5
Sierra Leone	2004	36.1	28.6	26.8	19.7
South Africa	2001	32.1	27.1	30.9	28.1
South Sudan	2008	34.7	28.5	32.9	20.4
Sudan	2008	34.5	28.3	27.9	21.7
Tanzania	2002	33.0	27.2	25.7	21.0
Uganda	2002	30.2	25.7	23.9	20.1

Source: Author computations from Census datasets

**Table 3:** Reproduced estimates from DHS reports based on a reference period of 0–6 years (_*35*_*q*_*15*_)

		Direct estimated _35_q_15_					
Country	DHS Survey Year	Male	95% CI	Female	95% CI	Total	95% CI
Cameroon	2011	0.2322	(0.2186–0.2459)	0.2282	(0.2151–0.2414)	0.2302	(0.2207–0.2397)
Kenya	2008/2009	0.2306	(0.2132–0.2480)	0.2143	(0.1982–0.2304)	0.2226	(0.2107–0.2344)
Liberia	2013	0.1508	(0.1359–0.1657)	0.1758	(0.1604–0.1912)	0.1638	(0.1531–0.1746)
Mali	2012/2013	0.1056	(0.0932–0.1180)	0.1005	(0.0878–0.1132)	0.1029	(0.0940–0.1117)
Sierra Leone	2013	0.1758	(0.1631–0.1884)	0.1815	(0.1693–0.1936)	0.1786	(0.1698–0.1874)
Tanzania	2010	0.1951	(0.1799–0.2103)	0.1959	(0.1808–0.2110)	0.1952	(0.1845–0.2059)
Uganda	2011	0.2530	(0.2338–0.2721)	0.2063	(0.1893–0.2233)	0.2294	(0.2166–0.2422)

Source: Author computations from DHS datasets

**Table 4:** Probability of dying between age 15 and 60 years (_45_*q*_15_) estimated from DHS sibling histories

		Directly estimated _45_q_15_						INDEPTH model smoothed estimated _45_q_15_	
Country	DHS Survey Year	Male	95% CI	Female	95% CI	Total	95% CI	Male	Female
Cameroon	2011	0.3943	(0.3723–0.4163)	0.3077	(0.2903–0.3251)	0.3517	(0.3377–0.3657)	0.5626	0.5696
Kenya	2008/2009	0.3814	(0.3543–0.4085)	0.2848	(0.2639–0.3057)	0.3348	(0.3177–0.3519)	0.5740	0.6043
Liberia	2013	0.2504	(0.2269–0.2739)	0.2586	(0.2367–0.2806)	0.2553	(0.2392–0.2714)	0.3744	0.4605
Mali	2012/2013	0.1629	(0.1441–0.1816)	0.1865	(0.1635–0.2094)	0.1735	(0.1589–0.1881)	0.2478	0.3088
Sierra Leone	2013	0.2577	(0.2397–0.2757)	0.2642	(0.2469–0.2814)	0.2610	(0.2486–0.2735)	0.3828	0.3923
Tanzania	2010	0.3215	(0.2974–0.3455)	0.2796	(0.2586–0.3006)	0.3012	(0.2853–0.3172)	0.4584	0.5604
Uganda	2011	0.4227	(0.3922–0.4532)	0.2920	(0.2688–0.3152)	0.3581	(0.3389–0.3772)	0.6197	0.5964

Source: Author computations from DHS datasets

*Reference period for the deaths 6 years before the survey

**Table 5:** Adult mortality estimates derived from sibling histories (_45_*q*_15_) by country and sex

	Females				Males			
	1990	1995	2000	2005	1990	1995	2000	2005
Mali	0.241	0.250	0.259	0.270	0.250	0.277	0.308	0.342
Cameroon	0.223	0.277	0.322	–	0.302	0.370	0.426	–
Kenya	0.182	0.268	0.347	–	0.223	0.316	0.397	–
Uganda	0.371	0.418	0.422	0.382	0.481	0.539	0.550	0.512
Tanzania	0.221	0.278	0.329	–	0.316	0.376	0.424	–
South Africa	0.103	0.171	–	–	0.286	0.369	–	–

Source: Reniers, G, B. Masquelier & P. Gerland.2011 in R. Rogers & E.M. Crimmins (eds), International Handbook of Adult Mortality

**Table 6:** United Nations Population Division adult mortality estimates (_45_*q*_15_) by country and sex

	Females				Males			
	1990	1995	2000	2005	1990	1995	2000	2005
Cameroon	0.305	0.352	0.399	0.381	0.355	0.389	0.421	0.401
Kenya	0.267	0.373	0.426	0.338	0.323	0.415	0.446	0.363
Liberia	0.287	0.265	0.325	0.262	0.363	0.283	0.365	0.301
Mali	0.318	0.332	0.310	0.282	0.313	0.325	0.299	0.275
Sierra Leone	0.537	0.544	0.491	0.440	0.572	0.589	0.525	0.455
South Africa	0.227	0.318	0.508	0.525	0.345	0.402	0.539	0.542
South Sudan	0.372	0.358	0.364	0.364	0.425	0.405	0.401	0.392
Sudan	0.269	0.258	0.242	0.223	0.325	0.325	0.314	0.287
Tanzania	0.373	0.432	0.438	0.360	0.422	0.461	0.449	0.373
Uganda	0.474	0.530	0.479	0.393	0.510	0.525	0.457	0.379

Source: United Nations, Department of Economic and Social Affairs, Population Division.2013. World Population Prospects: The 2012 Revision, DVD Edition

**Table 7:** World Health Organization adult mortality estimates (_45_*q*_15_) by country and sex

	Females				Males			
	1990	2000	2012	2013	1990	2000	2012	2013
Cameroon	0.287	0.375	0.349	0.341	0.340	0.398	0.371	0.370
Kenya	0.228	0.419	0.261	0.250	0.287	0.458	0.307	0.299
Liberia	0.376	0.336	0.246	0.240	0.544	0.375	0.282	0.279
Mali	0.343	0.361	0.277	0.275	0.348	0.364	0.282	0.277
Sierra Leone	0.512	0.521	0.426	0.423	0.525	0.551	0.444	0.444
South Africa	0.219	0.313	0.350	0.320	0.344	0.433	0.463	0.441
South Sudan	0.391	0.368	0.349	0.323	0.448	0.408	0.373	0.353
Sudan	0.276	0.249	0.214	0.212	0.342	0.318	0.276	0.274
Tanzania	0.328	0.427	0.277	0.244	0.388	0.454	0.342	0.314
Uganda	0.418	0.559	0.360	0.307	0.503	0.595	0.389	0.380

Source: http://apps.who.int/gho/indicatorregistry/Appp_Main/view_indicator.aspx?iid=64

## Footnotes

1 Visual assessment of population distribution plots by age and sex; age and sex ratio computations to assess misreporting and under reporting of age. Light smoothing was applied to gently modify irregularities in age structure without altering the totals.
